# Supplementary material for: Association of hepatic biomarkers with incident diabetes: a mediation analysis of the triglyceride-glucose index in a large Chinese cohort
Source: Lipids Health Dis. 2025 Jul 21;24:246. doi: 10.1186/s12944-025-02661-z (PMC12278604; doi:10.1186/s12944-025-02661-z)
Supplement: Supplementary file 1 — Supplementary Material 1 [file 12944_2025_2661_MOESM1_ESM.docx]

**Supplementary Materials Legend**

**Supplementary Table legend**

**Supplementary Table 1.** Baseline characteristics based on exclusion and inclusion participants.

**Supplementary Table 2.** univariate COX regression analysis.

**Supplementary Table 3.** Subgroup analysis of the relationship between ALT, AST, and ALT/AST and diabetes risk.

**Supplementary Table 4.** The predictive performance of ALT, AST, and the ALT/AST ratio for the incidence of diabetes at 3, 4, and 5 years.

**Supplementary Table 5.** Association of ALT, AST, and ALT/AST with TyG index.

**Supplementary Table 6.** E-values for mediation analysis.

**Supplementary Figure legend**

**Supplementary Figure 1.** Collinearity analysis.

**Supplementary Figure 2.** Proportional hazards assumption test of ALT.

**Supplementary Figure 3.** Proportional hazards assumption test of AST.

**Supplementary Figure 4.** Proportional hazards assumption test of the ALT/AST ratio.

**Supplementary Figure 5.** ROC for Tyg index predicting incidence of diabetes at 3, 4, and 5 years.

**Supplementary Figure 6.** The optimal cutoff values of ALT based on the maximum selected rank statistic.

**Supplementary Figure 7.** The optimal cutoff values of AST based on the maximum selected rank statistic.

**Supplementary Figure 8.** The optimal cutoff values of the ALT/AST ratio based on the maximum selected rank statistic.

**Supplementary Figure 9.** The optimal cutoff values of the TyG index based on the maximum selected rank statistic.

**Supplementary Table 1.** Baseline characteristics based on exclusion and inclusion participants.

| Variables | Exclude | Include | *P*-value |
| --- | --- | --- | --- |
|  | N = 161370 | N = 50463 |  |
| Age, years | 41.37 ± 12.37 | 44.41 ± 13.24 | <0.001 |
| Female, n (%) | 73369 (45.5) | 22341 (44.3) | <0.001 |
| BMI, kg/m^2^ | 23.17 ± 3.35 | 23.44 ± 3.30 | <0.001 |
| SBP, mm Hg | 118.83 ± 16.25 | 119.80 ± 16.78 | <0.001 |
| DBP, mm Hg | 74.08 ± 10.76 | 74.50 ± 10.99 | <0.001 |
| Smoking status, n (%) |  |  | <0.001 |
| Current | 9358 (5.8) | 2717 (5.4) |  |
| Ever | 1969 (1.2) | 590 (1.2) |  |
| Never | 35405 (21.9) | 10191 (20.2) |  |
| Not recorded | 114638 (71.0) | 36965 (73.3) |  |
| Drinking status, n (%) |  |  | <0.001 |
| Current | 908 (0.6) | 443 (0.9) |  |
| Ever | 6333 (3.9) | 2623 (5.2) |  |
| Never | 39491 (24.5) | 10432 (20.7) |  |
| Not recorded | 114638 (71.0) | 36965 (73.3) |  |
| Family history of diabetes, n (%) | 3249 (2.0) | 1095 (2.2) | 0.032 |
| FPG, mmol/L | 4.89 ± 0.61 | 5.01 ± 0.60 | <0.001 |
| Total cholesterol, mmol/L | 4.68 ± 0.90 | 4.78 ± 0.89 | <0.001 |
| Triglyceride, mmol/L | 1.05 (0.73, 1.60) | 1.10 (0.76, 1.67) | <0.001 |
| HDL-C, mmol/L | 1.37 ± 0.32 | 1.37 ± 0.30 | 0.487 |
| LDL-C, mmol/L | 2.77 ± 0.69 | 2.77 ± 0.67 | 0.073 |
| Blood urea nitrogen, mmol/L | 4.64 ± 1.18 | 4.71 ± 1.19 | <0.001 |
| Serum creatinine, mmol/L | 69.40 ± 15.43 | 72.09 ± 16.71 | <0.001 |
| ALT, U/L | 18.00 (12.90, 27.50) | 18.00 (13.00, 27.50) | <0.001 |
| AST, U/L | 21.80 (18.40, 26.20) | 22.00 (18.80, 27.00) | <0.001 |
| ALT/AST | 0.85 (0.66, 1.14) | 0.84 (0.65, 1.12) | <0.001 |
| TyG index | 8.36 ± 0.61 | 8.42 ± 0.61 | <0.001 |
| Diabetes | 2865 (1.8) | 1309 (2.6) | <0.001 |

Abbreviations: BMI, body mass index; SBP, systolic blood pressure; DBP, diastolic blood pressure; FPG, Fasting plasma glucose; HDL-C, high-density lipoprotein cholesterol; LDL-C, low-density lipoprotein cholesterol; ALT, alanine aminotransferase; AST, aspartate aminotransferase; TyG index, triglyceride-glucose index.

**Supplementary Table 2.** univariate COX regression analysis.

| **Variables** | HR (95% CI) | P-value |
| --- | --- | --- |
| Age, years | 1.06 (1.06, 1.07) | <0.001 |
| Female, n (%) | 0.60 (0.54, 0.68) | <0.001 |
| BMI, kg/m^2^ | 1.21 (1.20, 1.23) | <0.001 |
| SBP, mm Hg | 1.04 (1.04, 1.04) | <0.001 |
| DBP, mm Hg | 1.05 (1.04, 1.05) | <0.001 |
| Smoking status, n (%) |  |  |
| Current | Ref. |  |
| Ever | 0.86 (0.53, 1.39) | 0.5347 |
| Never | 0.48 (0.37, 0.61) | <0.001 |
| Not recorded | 0.69 (0.56, 0.85) | <0.001 |
| Drinking status, n (%) |  |  |
| Current | Ref. |  |
| Ever | 0.43 (0.25, 0.75) | 0.0031 |
| Never | 0.45 (0.27, 0.74) | 0.0019 |
| Not recorded | 0.52 (0.32, 0.86) | 0.0105 |
| Family history of diabetes, n (%) | 1.40 (1.03, 1.90) | 0.0334 |
| FPG, mmol/L | 9.48 (8.74, 10.28) | <0.001 |
| Total cholesterol, mmol/L | 1.30 (1.23, 1.37) | <0.001 |
| Triglyceride, mmol/L | 1.26 (1.24, 1.28) | <0.001 |
| HDL-C, mmol/L | 0.83 (0.69, 0.99) | 0.0367 |
| LDL-C, mmol/L | 1.28 (1.19, 1.38) | <0.001 |
| Blood urea nitrogen, mmol/L | 1.20 (1.16, 1.24) | <0.001 |
| Serum creatinine, mmol/L | 1.00 (1.00, 1.01) | <0.001 |
| ALT, U/L | 1.00 (1.00, 1.01) | <0.001 |
| AST, U/L | 1.01 (1.01, 1.01) | <0.001 |
| ALT/AST | 1.03 (1.02, 1.05) | <0.001 |
| TyG index | 3.69 (3.43, 3.97) | <0.001 |

Abbreviations: BMI, body mass index; SBP, systolic blood pressure; DBP, diastolic blood pressure; FPG, Fasting plasma glucose; HDL-C, high-density lipoprotein cholesterol; LDL-C, low-density lipoprotein cholesterol; ALT, alanine aminotransferase; AST, aspartate aminotransferase; TyG index, triglyceride-glucose index.

**Supplementary Figure 1.** Collinearity analysis.


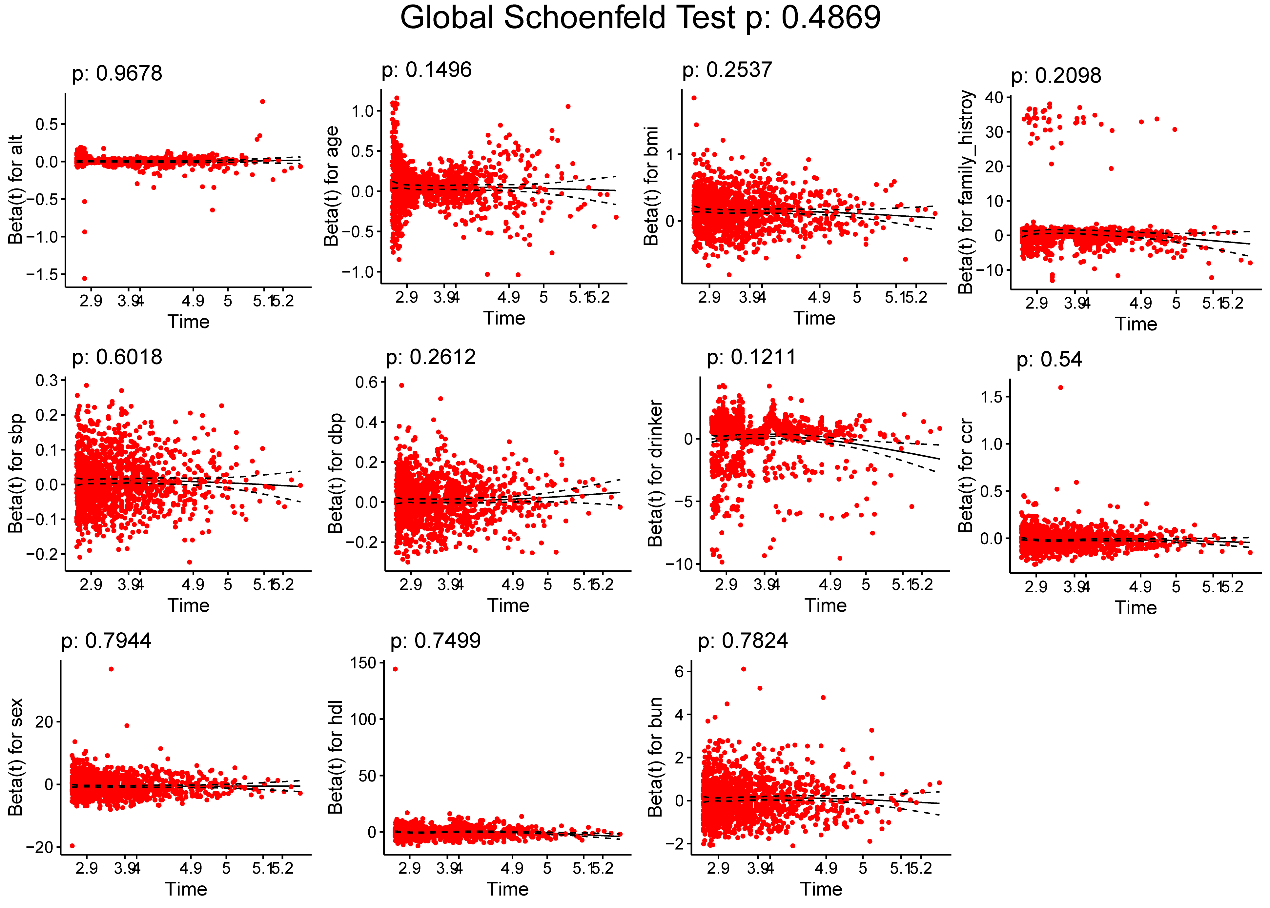


**Supplementary Figure 2.** Proportional hazards assumption test of ALT.


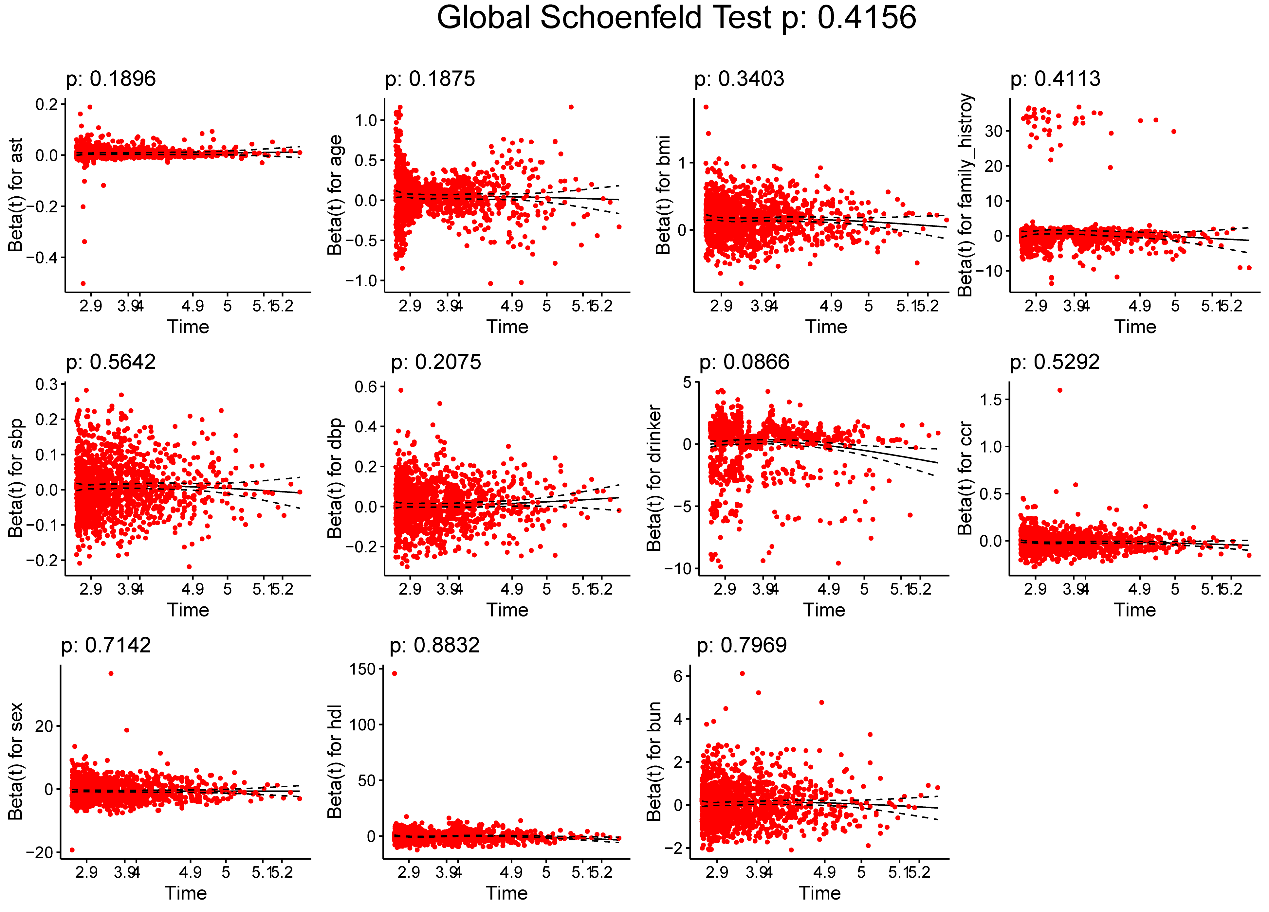


**Supplementary Figure 3.** Proportional hazards assumption test of AST.


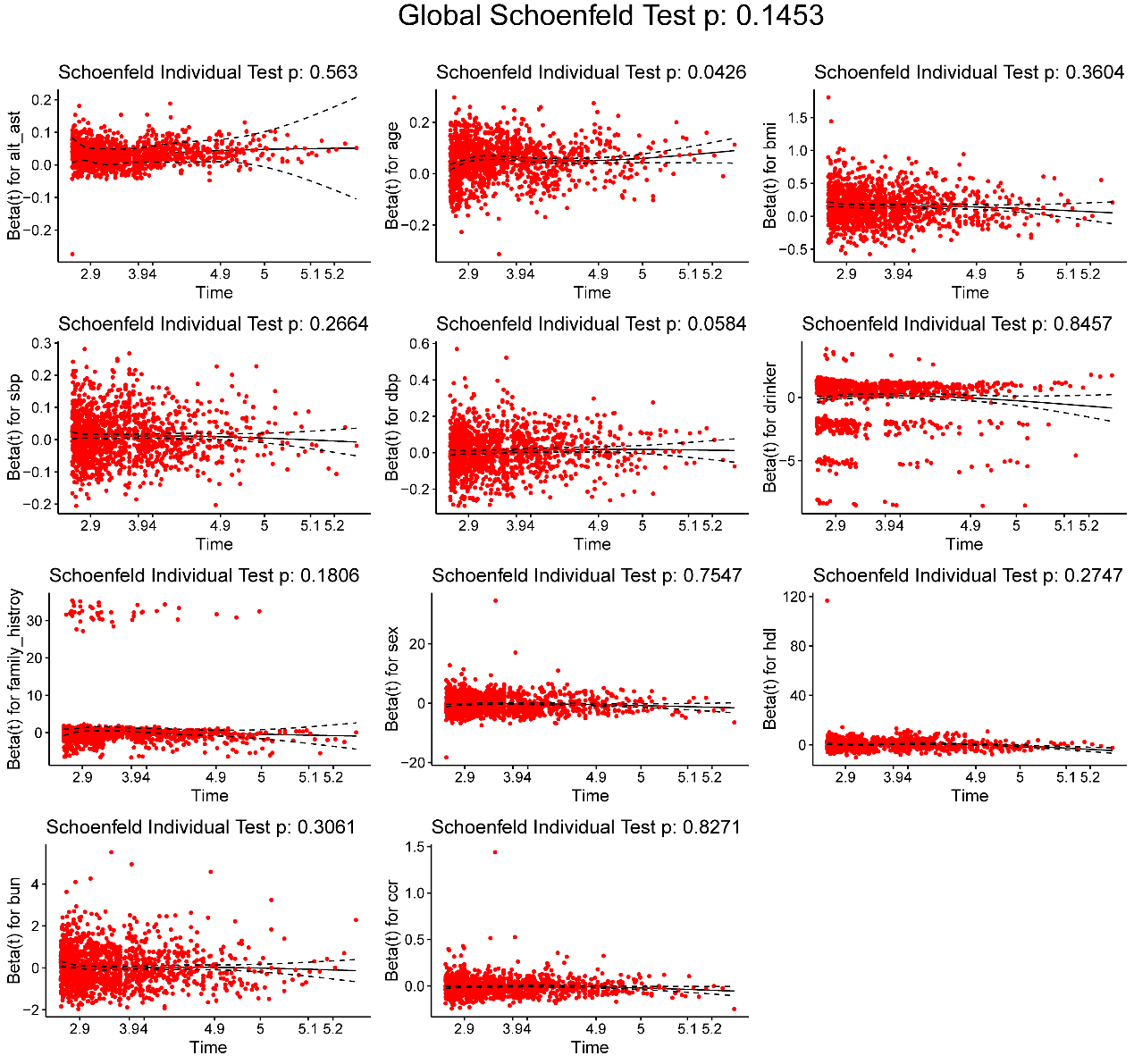


**Supplementary Figure 4.** Proportional hazards assumption test of the ALT/AST ratio.

**Supplementary Table 3.** Subgroup analysis of the relationship between ALT, AST, and ALT/AST and diabetes risk.

| Variables | HR 95% CI | *P*-value | *P* for interaction |
| --- | --- | --- | --- |
| **ALT** |  |  |  |
| Age, years |  |  | 0.48 |
| ≤60 | 1.00(1.00,1.00) | 0.51 |  |
| >60 | 1.00(1.00,1.01) | 0.51 |  |
| Sex |  |  | 0.002 |
| Male | 1.00(1.00,1.00) | 0.18 |  |
| Female | 1.01(1.00,1.01) | <0.001 |  |
| BMI, kg/m^2^ |  |  | 0.63 |
| ≤24 | 1.00(0.99,1.00) | 0.50 |  |
| >24 | 1.00(1.00,1.00) | 0.56 |  |
| Family history of diabetes | |  | 0.86 |
| No | 1.00(1.00,1.00) | 0.47 |  |
| Yes | 1.00(0.99,1.00) | 0.68 |  |
| **AST** |  |  |  |
| Age, years |  |  | 0.27 |
| ≤60 | 1.00(0.99,1.00) | 0.62 |  |
| >60 | 1.01(1.00,1.01) | 0.16 |  |
| Sex |  |  | 0.002 |
| Male | 1.00(0.99,1.00) | 0.20 |  |
| Female | 1.01(1.01,1.02) | <0.001 |  |
| BMI, kg/m^2^ |  |  | 0.77 |
| ≤24 | 1.00(0.99,1.01) | 0.74 |  |
| >24 | 1.00(0.99,1.00) | 0.86 |  |
| Family history of diabetes |  |  | 0.57 |
| No | 1.00(1.00,1.00) | 0.21 |  |
| Yes | 1.01(0.98,1.03) | 0.81 |  |
| **ALT/AST** |  |  |  |
| Age, years |  |  | 0.54 |
| ≤65 | 1.01(0.87,1.18) | 0.86 |  |
| >65 | 0.87(0.62,1.22) | 0.41 |  |
| Sex |  |  | 0.11 |
| Male | 0.95(0.82,1.11) | 0.55 |  |
| Female | 1.31(0.95,1.80) | 0.10 |  |
| BMI, kg/m^2^ |  |  | >0.99 |
| ≤24 | 0.96(0.72,1.27) | 0.76 |  |
| >24 | 0.96(0.83,1.11) | 0.58 |  |
| Family history of diabetes |  |  | 0.38 |
| No | 0.97(0.85,1.11) | 0.66 |  |
| Yes | 0.68(0.34,1.35) | 0.27 |  |

**Abbreviations:** ALT, alanine aminotransferase; AST, aspartate aminotransferase; BMI, body mass index; HR Hazard ratio; CI confidence interval

**Supplementary Table 4.** The predictive performance of ALT, AST, and the ALT/AST ratio for the incidence of diabetes at 3, 4, and 5 years.

| Variables | AUC | 95% CI | Sensitivity | Specificity | Youden index |
| --- | --- | --- | --- | --- | --- |
| ALT |  |  |  |  |  |
| 3-year | 0.64 | 0.62-0.65 | 0.70 | 0.50 | 0.20 |
| 4-year | 0.63 | 0.61-0.65 | 0.69 | 0.51 | 0.20 |
| 5-year | 0.63 | 0.61-0.67 | 0.44 | 0.75 | 0.18 |
| AST |  |  |  |  |  |
| 3-year | 0.59 | 0.57-0.62 | 0.40 | 0.76 | 0.17 |
| 4-year | 0.64 | 0.63-0.66 | 0.74 | 0.48 | 0.22 |
| 5-year | 0.61 | 0.58-0.64 | 0.44 | 0.75 | 0.18 |
| ALT/AST ratio |  |  |  |  |  |
| 3-year | 0.63 | 0.60-0.65 | 0.55 | 0.66 | 0.21 |
| 4-year | 0.59 | 0.57-0.61 | 0.64 | 0.52 | 0.17 |
| 5-year | 0.61 | 0.59-0.64 | 0.64 | 0.54 | 0.18 |
| TyG index |  |  |  |  |  |
| 3-year | 0.77 | 0.75-0.79 | 0.81 | 0.62 | 0.43 |
| 4-year | 0.77 | 0.76-0.78 | 0.78 | 0.64 | 0.42 |
| 5-year | 0.78 | 0.76-0.81 | 0.84 | 0.61 | 0.44 |

**Supplementary Table 5.** Association of ALT, AST, and ALT/AST with TyG index.

| **Model** | **Model 1** | ***P*-value** | **Model 2** | ***P*-value** | **Model 3** | ***P*-value** |
| --- | --- | --- | --- | --- | --- | --- |
|  | **β (95%CI)** |  | **β (95%CI)** |  | **β (95%CI)** |  |
| **TyG index ~ ALT** |  |  |  |  |  |  |
| ALT | 0.0034(0.0031, 0.0036) | <0.001 | 0.0033(0.0031, 0.0036) | <0.001 | 0.0033(0.0031, 0.0036) | <0.001 |
| Quartile of ALT |  |  |  |  |  |  |
| Q1 | Ref. |  | Ref. |  | Ref. |  |
| Q2 | 0.0616(0.0487, 0.0746) | <0.001 | 0.0615(0.0485, 0.0744) | <0.001 | 0.0657(0.0529, 0.0785) | <0.001 |
| Q3 | 0.1644(0.1508, 0.1779) | <0.001 | 0.164(0.1504, 0.1776) | <0.001 | 0.1665(0.1531, 0.1799) | <0.001 |
| Q4 | 0.3248(0.3101, 0.3394) | <0.001 | 0.3237(0.3091, 0.3384) | <0.001 | 0.3252(0.3107, 0.3397) | <0.001 |
| *P* for trend |  | <0.001 |  | <0.001 |  | <0.001 |
| **TyG index ~ AST** |  |  |  |  |  |  |
| AST | 0.0033(0.0029, 0.0037) | <0.001 | 0.0033(0.0029, 0.0036) | <0.001 | 0.0033(0.0030, 0.0037) | <0.001 |
| Quartile of AST |  |  |  |  |  |  |
| Q1 | Ref. |  | Ref. |  | Ref. |  |
| Q2 | 0.0279(0.0151, 0.0407) | <0.001 | 0.0271(0.0143, 0.0399) | <0.001 | 0.0298(0.0171, 0.0424) | <0.001 |
| Q3 | 0.0891(0.0760, 0.1023) | <0.001 | 0.0877(0.0745, 0.1009) | <0.001 | 0.093(0.0800, 0.1060) | <0.001 |
| Q4 | 0.1809(0.1670, 0.1949) | <0.001 | 0.1785(0.1645, 0.1924) | <0.001 | 0.1869(0.1731, 0.2007) | <0.001 |
| *P* for trend |  | <0.001 |  | <0.001 |  | <0.001 |
| **TyG index ~ ALT/AST ratio** | |  |  |  |  |  |
| ALT/AST ratio | 0.0895(0.0820, 0.0970) | <0.001 | 0.0899(0.0824, 0.0973) | <0.001 | 0.0889(0.0815, 0.0963) | <0.001 |
| Quartile of ALT/AST ratio |  |  |  |  |  |  |
| Q1 | Ref. |  | Ref. |  | Ref. |  |
| Q2 | 0.056(0.0432, 0.0688) | <0.001 | 0.0576(0.0448, 0.0704) | <0.001 | 0.0554(0.0428, 0.0681) | <0.001 |
| Q3 | 0.1526(0.1392, 0.1659) | <0.001 | 0.1545(0.1411, 0.1678) | <0.001 | 0.1507(0.1375, 0.1640) | <0.001 |
| Q4 | 0.2928(0.2780, 0.3075) | <0.001 | 0.2945(0.2798, 0.3093) | <0.001 | 0.2889(0.2743, 0.3036) | <0.001 |
| *P* for trend |  | <0.001 |  | <0.001 |  | <0.001 |

Model 1: Adjusted for age, sex, BMI, SBP, DBP at baseline. Model 2: Further adjusted for drinking status, family history of diabetes based on model 1. Model 3: Further adjusted for HDL-C, SCr and BUN based on model 2.

Abbreviations: ALT, alanine aminotransferase; AST, aspartate aminotransferase; TyG index, triglyceride-glucose index; BMI, body mass index; SBP, systolic blood pressure; DBP, diastolic blood pressure; SCr, Serum creatinine; BUN, Blood urea nitrogen; HDL-C high-density lipoprotein cholesterol, HR Hazard ratio; CI confidence interval

**
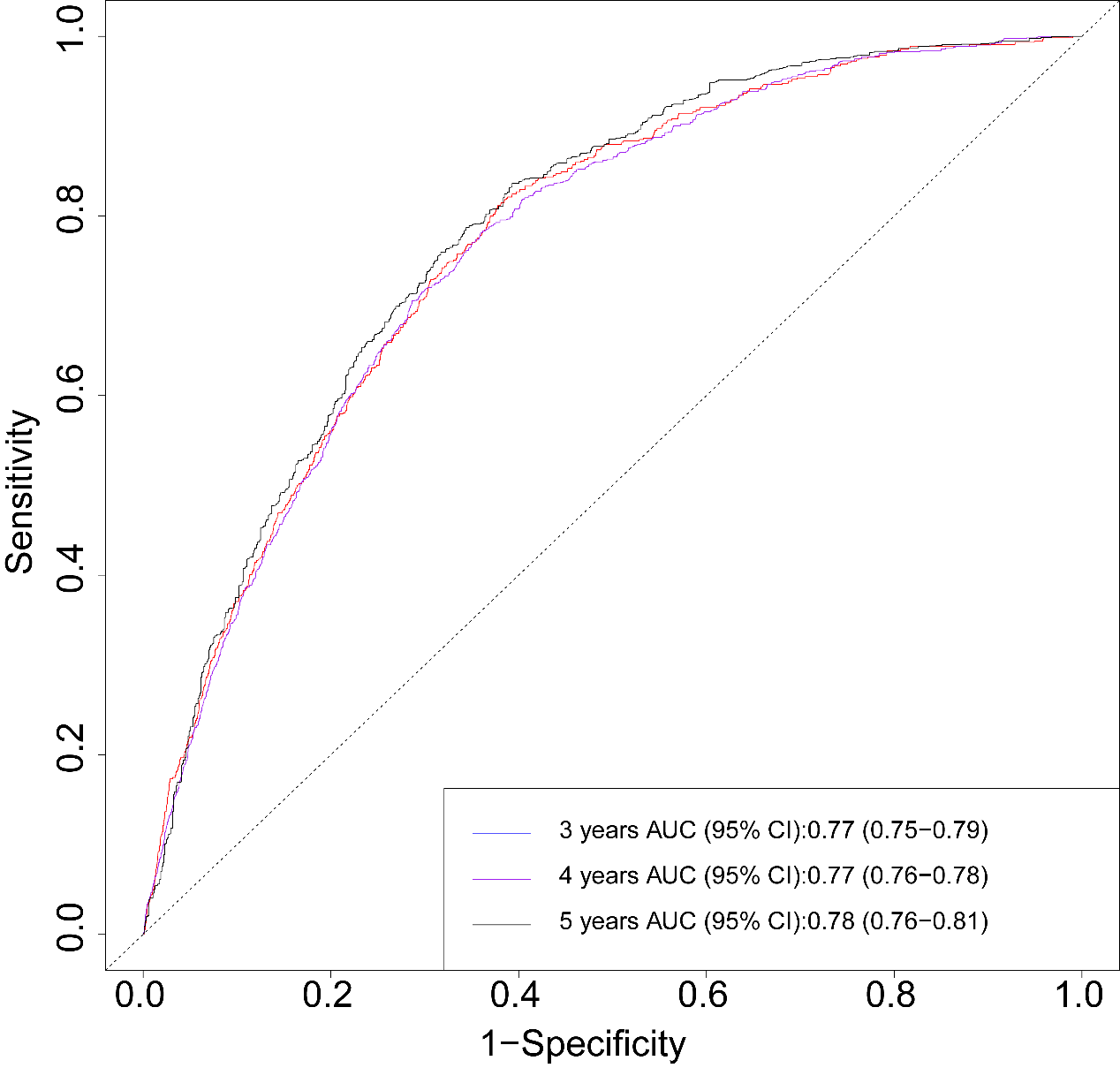
**

**Supplementary Figure 5.** ROC for Tyg index predicting incidence of diabetes at 3, 4, and 5 years.


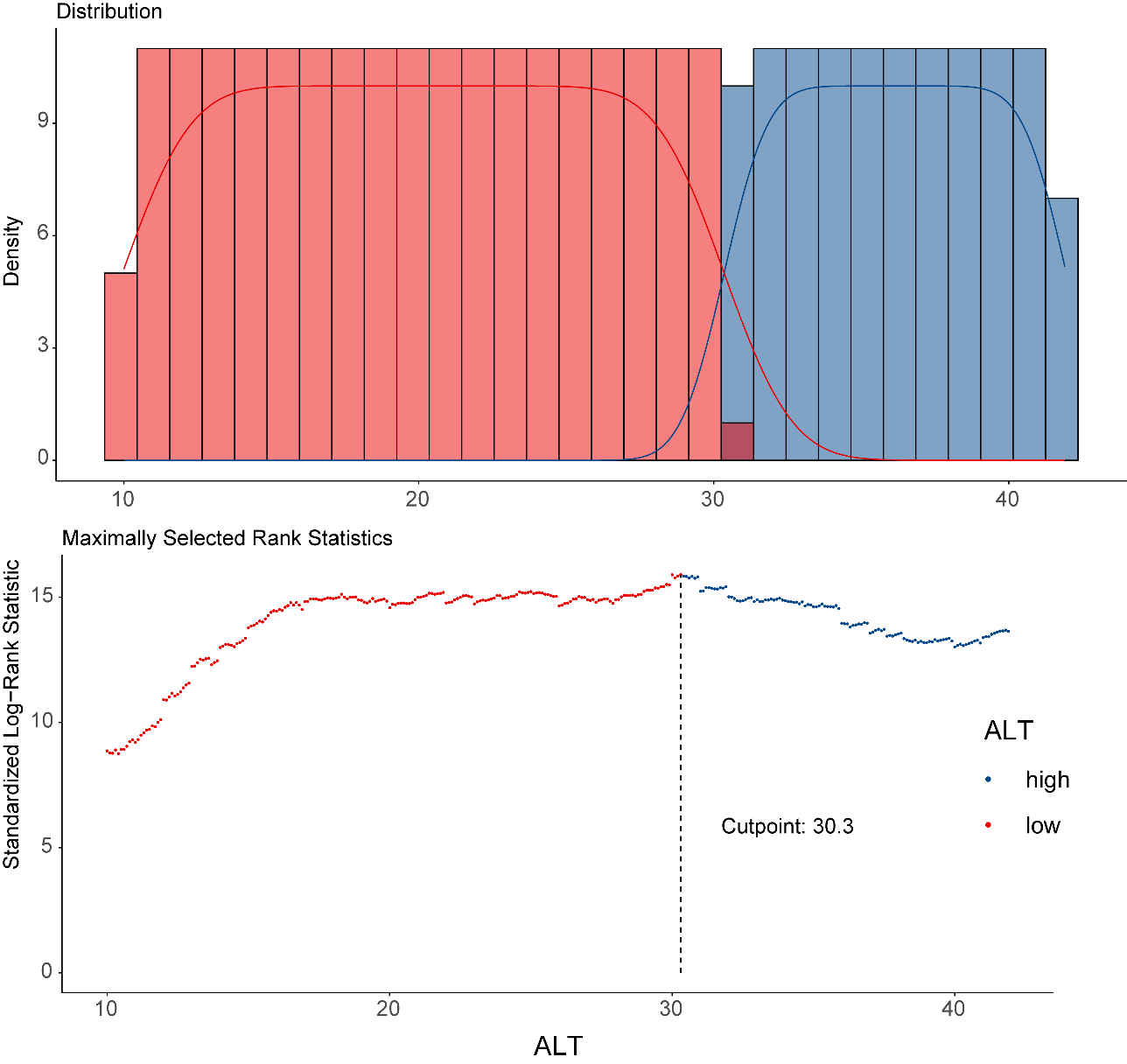


**Supplementary Figure 6.** The optimal cutoff values of ALT based on the maximum selected rank statistic.


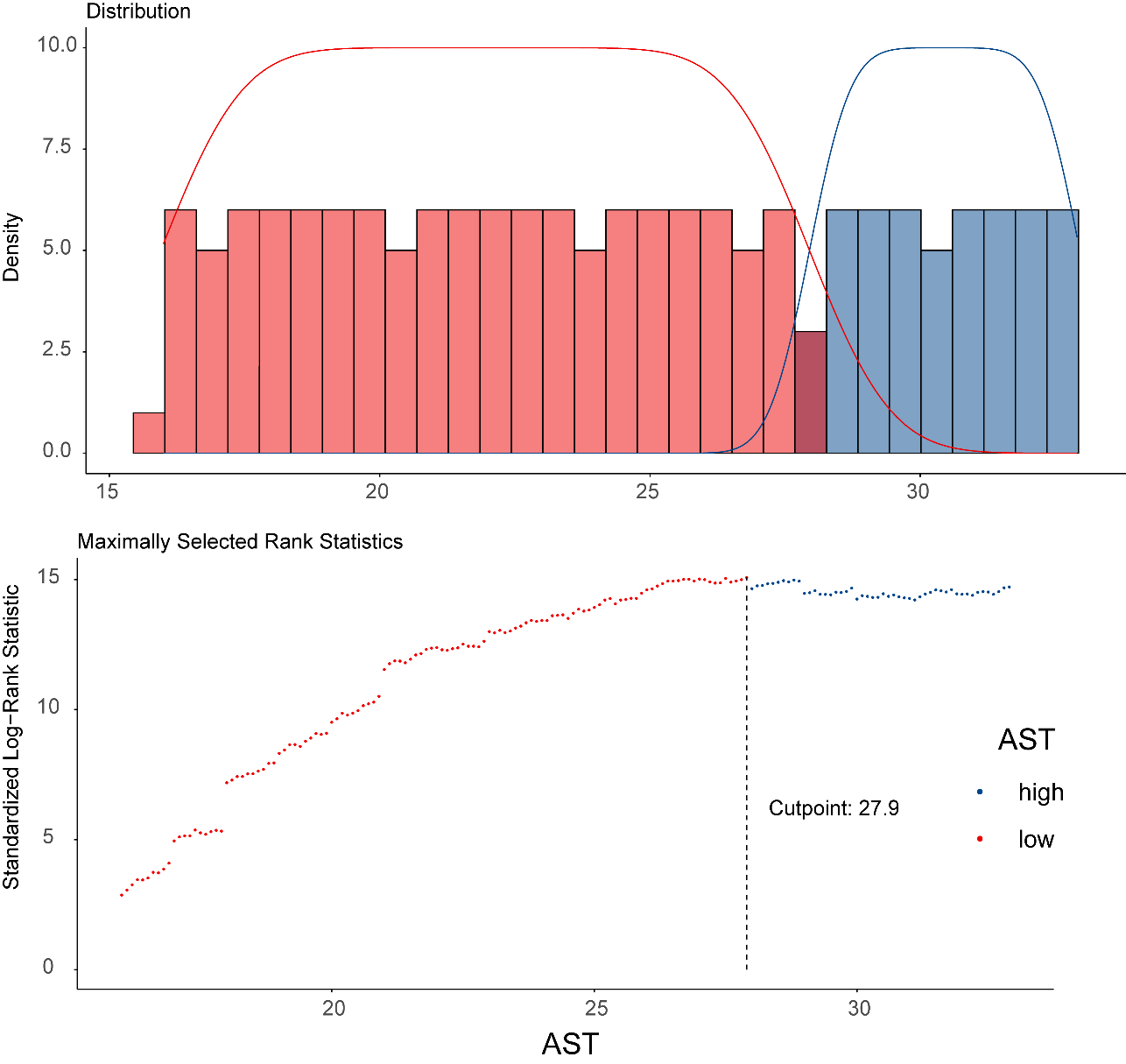


**Supplementary Figure 7.** The optimal cutoff values of AST based on the maximum selected rank statistic.


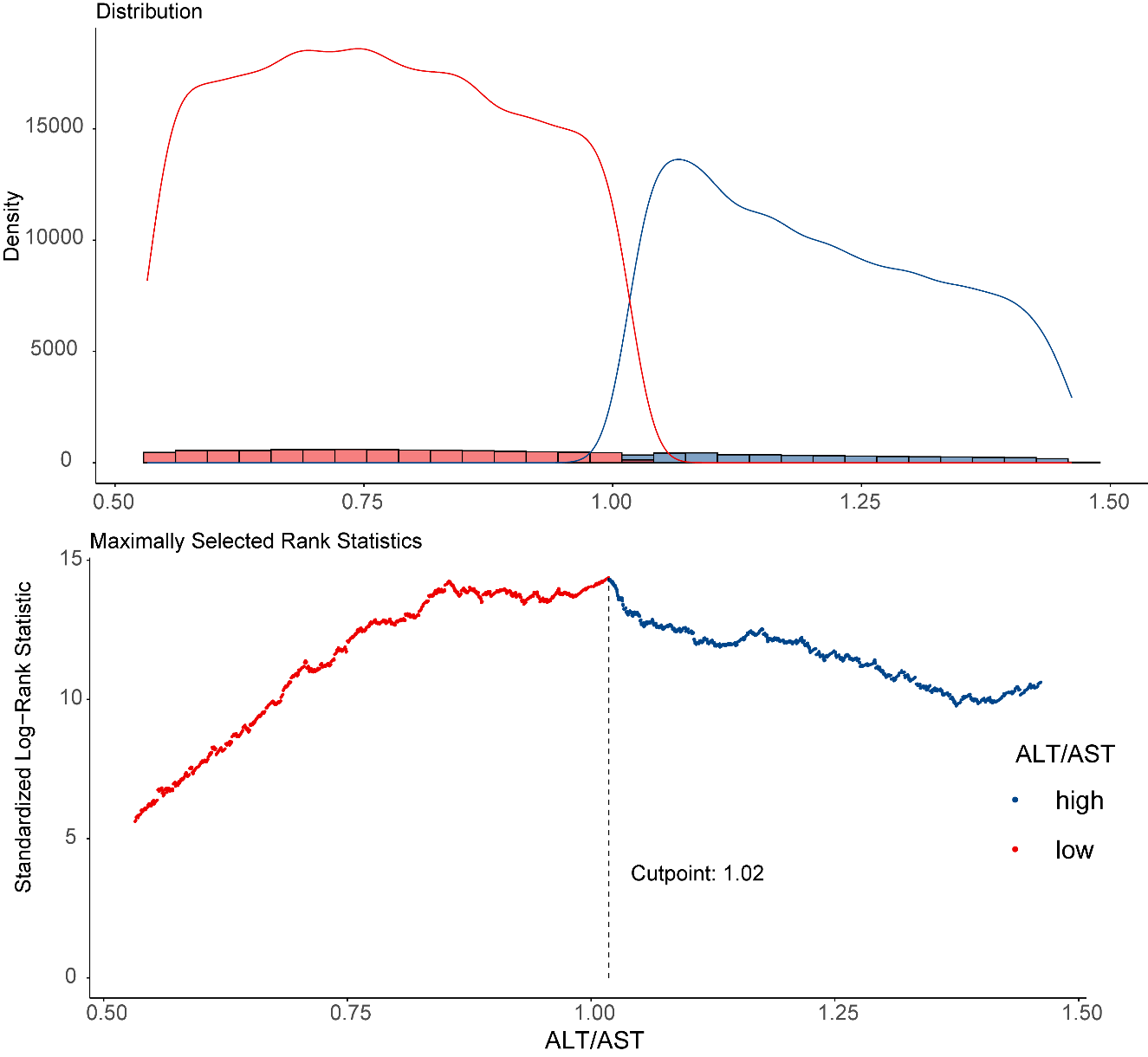


**Supplementary Figure 8.** The optimal cutoff values of the ALT/AST ratio based on the maximum selected rank statistic.


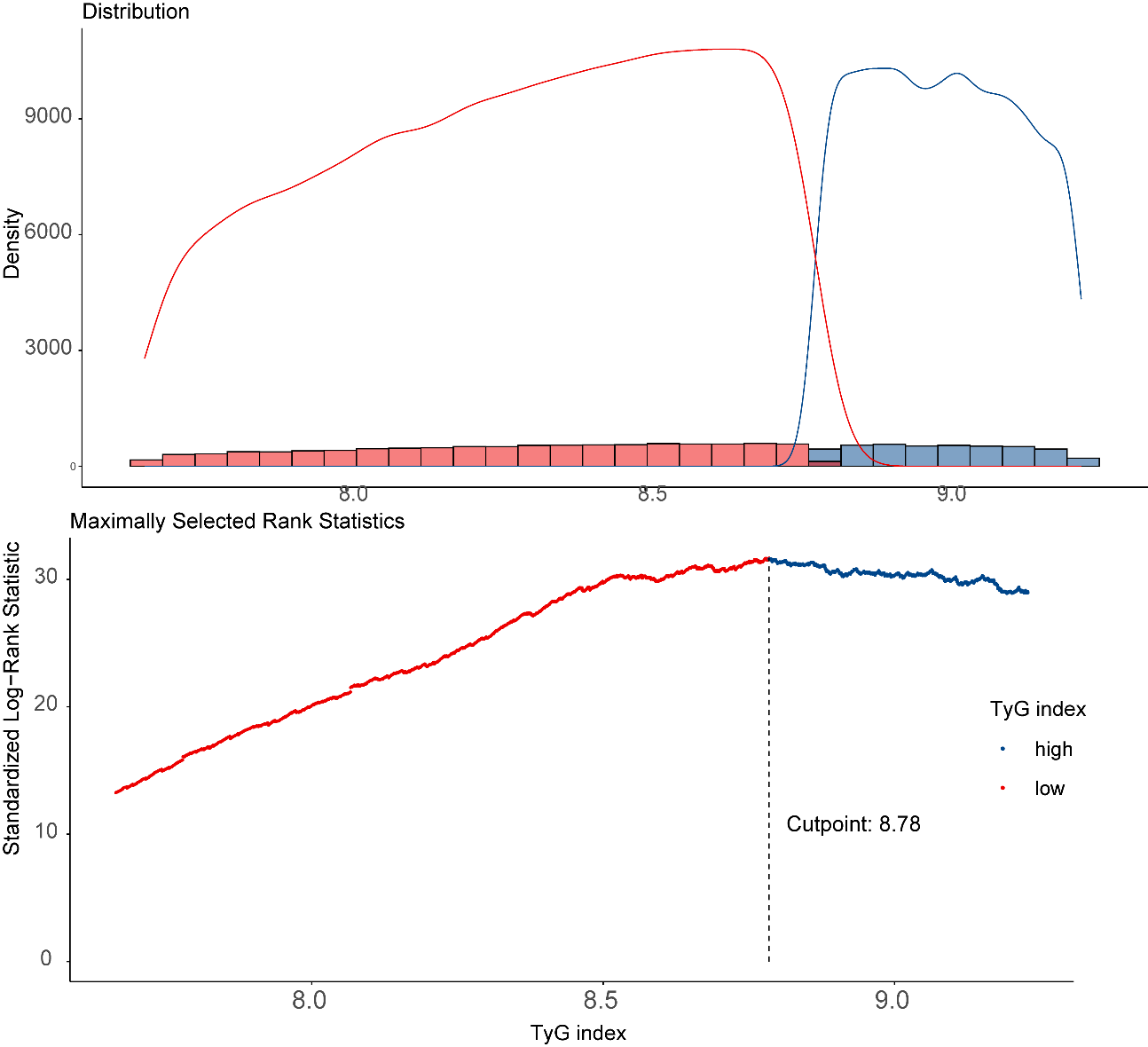


**Supplementary Figure 9.** The optimal cutoff values of the TyG index based on the maximum selected rank statistic.

**Supplementary Table 6.** E-values for mediation analysis.

| **Variables** | **E-value (Total Effect)** | **E-value (Direct Effect)** | **E-value (Indirect Effect)** |
| --- | --- | --- | --- |
| ALT, U/L | 1.10 | 1.07 | 1.06 |
| AST, U/L | 1.10 | 1.08 | 1.06 |
| ALT/AST ratio | 1.49 | 1.19 | 1.41 |
