# Supplementary material for: Association of hepatic biomarkers with incident diabetes: a mediation analysis of the triglyceride-glucose index in a large Chinese cohort
Source: Lipids Health Dis. 2025 Jul 21;24:246. doi: 10.1186/s12944-025-02661-z (PMC12278604; doi:10.1186/s12944-025-02661-z)
Supplement: Supplementary file 2 — Supplementary Material 2 [file 12944_2025_2661_MOESM2_ESM.pdf]

924385142997254144.docx

University of Chittagong

Document Details

Submission ID

trn:oid:::3618:102668125

Submission Date

Jun 27, 2025, 12:26 PM GMT+8

Download Date

Jun 27, 2025, 12:29 PM GMT+8

File Name

924385142997254144.docx

File Size

99.4 KB

21 Pages

4,787 Words

28,382 Characters

14% Overall Similarity

The combined total of all matches, including overlapping sources, for each database.

Filtered from the Report

- Bibliography

Match Groups

84 Not Cited or Quoted 14%
Matches with neither in-text citation nor quotation marks

1 Missing Quotations 0%
Matches that are still very similar to source material

0 Missing Citation 0%
Matches that have quotation marks, but no in-text citation

0 Cited and Quoted 0%
Matches with in-text citation present, but no quotation marks

Top Sources

8% Internet sources

10% Publications

0% Submitted works (Student Papers)

Integrity Flags

0 Integrity Flags for Review

No suspicious text manipulations found.

Our system's algorithms look deeply at a document for any inconsistencies that would set it apart from a normal submission. If we notice something strange, we flag it for you to review.

A Flag is not necessarily an indicator of a problem. However, we'd recommend you focus your attention there for further review.

Match Groups

- **84** Not Cited or Quoted 14%
  Matches with neither in-text citation nor quotation marks
- **1** Missing Quotations 0%
  Matches that are still very similar to source material
- **0** Missing Citation 0%
  Matches that have quotation marks, but no in-text citation
- **0** Cited and Quoted 0%
  Matches with in-text citation present, but no quotation marks

Top Sources

8% Internet sources
10% Publications
0% Submitted works (Student Papers)

Top Sources

The sources with the highest number of matches within the submission. Overlapping sources will not be displayed.

1	Internet	www.frontiersin.org	1%
2	Publication	Yanyan Xuan, Dingting Wu, Qin Zhang, Zhiqiang Yu, Jingbo Yu, Dongdong Zhou. "...	<1%
3	Publication	Fang-Chen Liu, Kai-Wen Chen, Kun-Zhe Tsai, Chen-Chih Chu, Yen-Chen Lin, Yun-Ch...	<1%
4	Internet	www.mdpi.com	<1%
5	Publication	Yangchun Wang, Fei Liu, Ruixiang Tong, Zhonghua He, Qin Fang, Jie Feng, Hongli...	<1%
6	Publication	Qiong Li, Jing Yan, Chenyang Zhao, Meng Li, Ying Gu, Bingshun Wang, Yangjing P...	<1%
7	Internet	www.science.gov	<1%
8	Internet	science.gov	<1%
9	Publication	Jiashu Yao, Wei Li, Yu Liu, Tongzhi Wu, Duolao Wang, Radivoj Arezina, Zilin Sun, S...	<1%
10	Publication	Mohammadi, Fatemeh, Mostafa Qorbani, Roya Kelishadi, Fereshteh Baygi, Gelayo...	<1%

11	Publication	
"Posters", Hepatology, 2020		<1%
12	Publication	
Boyi Yang, Shujun Fan, Xueyuan Zhi, Jing He, Ping Ma, Luyang Yu, Quanmei Zhen...		<1%
13	Publication	
Diego Elias da Silva Caldeira, Marina Rodrigues Garcia da Silveira, Maria Rita Rodr...		<1%
14	Publication	
Kawamoto, Ryuichi, Katsuhiko Kohara, Tomo Kusunoki, Yasuharu Tabara, Masan...		<1%
15	Internet	
bmjopen.bmj.com		<1%
16	Publication	
Saely, C.H.. "Alanine aminotransferase and gamma-glutamyl transferase are asso...		<1%
17	Publication	
Yanhua Hu, Yong Han, Yufei Liu, Yanan Cui, Zhiping Ni, Ling Wei, Changchun Cao,...		<1%
18	Internet	
bmcendocrdisord.biomedcentral.com		<1%
19	Publication	
Jinjin Jian, Dongxiao Huang, Jinfang Zeng. "The association between red blood cel...		<1%
20	Publication	
Anran Wang, Yapeng Li, Lue Zhou, Kai Liu et al. "Triglyceride-Glucose Index Is Rel...		<1%
21	Publication	
Yongfang Zhang, Jiahao Song, Ming Zhang, Gaoyin Xiong et al. "Arsenic exposure,...		<1%
22	Internet	
www.xiahepublishing.com		<1%
23	Internet	
cran.ms.unimelb.edu.au		<1%
24	Internet	
www.dovepress.com		<1%

25 Publication

Guangli Yang, Siyuan Zhang, Yanhe Wang, Bingyu Han, Dongsheng Sun. "Associat... <1%

26 Internet

jaha.ahajournals.org <1%

27 Publication

Huang Yu, Tingyi Zhang, Yankun Liu, Wang Wang, Ziyi Guan, Ping Li. "Association ... <1%

28 Internet

ul.qucosa.de <1%

29 Internet

www.biomedcentral.com <1%

30 Publication

Heqing Tao, Ligang Liu, Weipeng Lu, Ziyan Ni, Xueqing Chen, Milap C. Nahata, Lia... <1%

31 Internet

link.springer.com <1%

32 Internet

nutritionj.biomedcentral.com <1%

33 Internet

onlinelibrary.wiley.com <1%

34 Internet

pubmed.ncbi.nlm.nih.gov <1%

35 Internet

1library.net <1%

36 Publication

Du, Tingting, Gang Yuan, Muxun Zhang, Xinrong Zhou, Xingxing Sun, and Xuefen... <1%

37 Publication

Lei Zhao, Runqing Mu, Xin Zhang, Shuo Wang, Min Zhao, Hong Shang. "Age-Relat... <1%

38 Publication

N. Sattar. "Biomarkers for diabetes prediction, pathogenesis or pharmacotherap... <1%

39 Publication

Xuecheng Zhao, Enmin Xie, Zhengqin Zhai, Di Sun, Siqi Shen, Haoming He, Wei Liu... <1%

40 Internet

downloads.hindawi.com <1%

41 Internet

pure.eur.nl <1%

42 Internet

repub.eur.nl <1%

43 Internet

worldwidescience.org <1%

44 Internet

www.jpmph.org <1%

45 Internet

www.ncbi.nlm.nih.gov <1%

46 Publication

Giuseppe Mancia, Guido Grassi, Konstantinos P. Tsioufis, Anna F. Dominiczak, Enr... <1%

47 Publication

M. C. Devers. "Should liver function tests be included in definitions of metabolic s... <1%

48 Publication

Park, So Yun, Yeon Jean Cho, Sa Ra Lee, Hyewon Chung, and Kyungah Jeong. "Trig... <1%

49 Publication

"Cirrhosis and Portal Hypertension (Screening Diagnosis & Treatment)", Journal o... <1%

Association of Hepatic Biomarkers with Incident Diabetes: A Mediation Analysis of the Triglyceride-Glucose Index in a Large Chinese Cohort

Abstract

Background: Diabetes disproportionately impacts low- and middle-income populations, exacerbating existing health disparities. The role of hepatic biomarkers, including aspartate aminotransferase (AST), alanine aminotransferase (ALT), and the ALT/AST ratio, in predicting diabetes onset remains insufficiently elucidated. This research assessed how these biomarkers relate to diabetes risk, as well as assessed the mediating effect of the triglyceride-glucose (TyG) index.

Methods: The secondary analysis utilized data from the Dryad public database, encompassing a cohort of 211833 Chinese adults aged ≥20 years who underwent health examinations between 2010 and 2016. After applying rigorous exclusion criteria, 50463 participants were included. Cox proportional hazards models were applied to examine how hepatic biomarkers and the TyG index influenced diabetes incidence. The mediation analysis was conducted to assess the TyG index's contribution to the hepatic biomarker-diabetes relationship.

Results: Throughout the observational phase (mean 3.08 years), 1309 participants (2.59%) established diabetes. Increased levels of ALT, AST, and the ALT/AST ratio were all significantly related to a heightened diabetes risk, with the most significant

correlation noted for the ALT/AST ratio (adjusted HR per unit increase: 1.04; 95% CI: 1.02–1.05; $P < 0.001$). Participants in the highest quartile of the ALT/AST ratio had nearly three times the risk of diabetes than the lowest quartile (HR: 2.94; 95% CI: 2.42–3.57; $P < 0.001$). Joint analysis revealed synergistic effects between elevated hepatic biomarkers and the TyG index, with the combination of high ALT/AST ratio and elevated TyG index yielding the greatest risk (HR: 5.23; 95% CI: 4.42–6.18; $P < 0.001$). The mediation analysis showed that the TyG index significantly mediated the associations, accounting for 40.25%, 36.45%, and 76.97% of the effects of ALT, AST, and the ALT/AST ratio, respectively, on diabetes risk.

Conclusion: Hepatic biomarkers, particularly the ALT/AST ratio, robustly predict diabetes risk in this large cohort, with the TyG index explaining most of this association. These insights reinforce the importance of integrating hepatic and metabolic assessment in preventive strategies to address the growing diabetes epidemic.

Keywords: hepatic biomarkers; triglyceride-glucose index; diabetes; incidence; mediation analysis

Introduction

The global prevalence of diabetes is increasing rapidly worldwide, affecting an estimated 537 million adults in 2021. By 2045, this number is forecasted to reach 783 million [1]. The rapid expansion of this epidemic presents a substantial public health concern, given that diabetes significantly drives the burden of cardiovascular disease,

chronic kidney disease, and early death [2]. It is expected that the diabetes prevalence rate among adults aged 20 to 79 in China will increase from 8.2% to 9.7% during 2020-2030, while the share of healthcare costs relative to GDP will rise from 1.58% to 1.69% [3]. Despite advancements in prevention and management, the prevalence of diabetes continues to climb, particularly in low- and middle-income countries, where urbanization and aging populations exacerbate the risk [4]. Therefore, early detection of high diabetes-risk individuals enables targeted interventions and alleviates the social and economic burdens of the disease.

Previous research highlights the potential role of hepatic biomarkers, particularly alanine aminotransferase (ALT), aspartate aminotransferase (AST), and the ALT/AST ratio, in predicting diabetes risk [5]. These enzymes, traditionally linked to liver damage, are increasingly recognized as indicators of metabolic dysregulation [6-8]. Elevated ALT levels, for example, are strongly linked to hepatic steatosis and systemic inflammation, both of which are implicated in the pathogenesis of insulin resistance and diabetes [9, 10]. The ALT/AST ratio is also a reliable marker of insulin resistance across various populations [11-13]. Nevertheless, several critical gaps remain in the literature. First, although the ALT/AST ratio is associated with insulin resistance, its utility in forecasting incident diabetes has not been thoroughly investigated. Second, while the triglyceride-glucose (TyG) index has gained recognition as a predictor of cardiometabolic outcomes [14-16], its role in the hepatic biomarker-diabetes pathway remains inadequately understood. Finally, despite the unique metabolic profiles and

higher prevalence of insulin resistance in Asian populations, large-scale studies exploring these associations in Asian cohorts are limited.

This study aims to comprehensively examine the associations between hepatic biomarkers (ALT, AST, and the ALT/AST ratio) and the development of diabetes among 50463 Chinese adults, while also exploring the TyG index's mediating role in these associations.

Methods

Study populations

The data obtained from the Dryad public database (https://datadryad.org/), an internationally renowned open-access data repository that adheres to the principles of open science, enabling researchers to freely access datasets for secondary analyses to explore novel research hypotheses. This dataset used for this study encompasses a comprehensive cohort of 211833 Chinese adults aged 20 years and older who underwent health examinations between 2010 and 2016 across 32 sites in 11 cities in China. All participants had at least two clinical visits during the follow-up period. The dataset was originally proposed by Chen et al [17]. The Rich Healthcare Group Institutional Review Board approved the original study. Consent requirements were waived, as the study involved only secondary analysis of existing data.

The data originally included 685277 participants, 473444 participants were excluded

from the Chen et al. study, and finally 211833 participants were analyzed in the original study. Exclusion criteria: (1) Participants lacking baseline height and weight measurements (n=103946); (2) Participants with extreme body mass index (BMI) (<15 kg/m^2 or >55 kg/m^2) (n=152); (3) Participants without gender information (n=1) at baseline; (4) Participants with a pre-existing diabetes diagnosis at enrollment (n=7112); (5) Participants with missing fasting plasma glucose (FPG) at baseline (n=31370); (6) Participants with undetermined diabetes status at follow-up (6630); (7) Participants with < 2 years of follow-up (n=324233). Additionally, based on the purpose of this study, the additional exclusion criteria were set: (1) participants with missing ALT, AST, and the ALT/AST ratio at baseline (n = 123951); (2) participants with missing baseline TyG index at baseline (n = 1706); (3) participants with missing low-density lipoprotein cholesterol (LDL-C) and high-density lipoprotein cholesterol (HDL-C) at baseline (n = 35713). The final study cohort comprised 50463 eligible participants. In addition, the present study also added baseline characteristics based on exclusion and inclusion participants (**Supplementary Table 1**). All procedures and methodologies conformed to the ethical principles of the Declaration of Helsinki.

Clinical data collection

At each visit, participants completed a standardized questionnaire on sociodemographic data, including age, sex, lifestyle (alcohol and smoking), and family history of diabetes. Based on baseline time, smoking and drinking status were categorized as none, former, current, and not recorded. Trained examiners measured blood pressure when

participants were at calm using a standard mercury sphygmomanometer. Participants' weight and weight were assessed in lightweight attire and without footwear, and recorded to 0.1 kg and 0.1 cm, respectively. BMI was calculated as weight (kg) / height^2 (m^2).

Participants had fasted for at least 10 hours when venous blood was collected. hepatic biomarkers contained ALT, AST, and the ALT/AST ratio; kidney function biomarkers included serum creatinine (SCr) and blood urea nitrogen (BUN); Lipid parameters consisted of total cholesterol (TC), HDL-C, LDL-C, and triglyceride (TG); The above blood indicators were measured using an automatic biochemical analyzer (Beckman Coulter AU5800, Brea, California, USA). PFG were measured by the glucose oxidase method. The ALT/AST ratio = ALT/AST; TyG index = ln [TG (mg/dL) × FPG (mg/dL)/2] [14].

Diagnosis of diabetes

diabetes, as a primary outcome, was identified as either self-reported physician-diagnosed diabetes during follow-up or FPG levels meeting or exceeding the diagnostic threshold of 7.00 mmol/L (126 mg/dL) at follow-up visits. The event date was defined as the earliest date of diabetes diagnosis, identified through the ascertainment method, or the date of the final follow-up visit, whichever occurred first.

Statistical analysis

Statistical analyses were conducted utilizing R program (version 4.4.0). Normally distributed continuous variables are reported as mean ± SD, non-normal as median (IQR), and categorical variables as n (%). Continuous variables followed t-tests (normal) or Mann-Whitney U (non-normal); chi-square tests assessed categorical variables.

Initially, treatment of missing values: Continuous variables such as SBP (missing percentage: 0.02%), DBP (missing percentage: 0.02%), HDL-C (missing percentage: 1.86%), LDL-C (missing percentage: 0.19%), BUN (missing percentage: 3.13%), and serum creatinine (missing percentage: 1.26%) were imputed with multiple imputation. Unavailable entries for categorical variables, including smoking and drinking habits, were designated as not documented

Next, hepatic biomarkers were evaluated for diabetes risk using Cox regression, presented as hazard ratios (HRs) (95% confidence intervals, CIs). Three multivariate adjustment models were constructed: Model 1: Adjusted for age, sex, BMI, SBP, DBP at baseline. Model 2: Further adjusted for drinking status, family history of diabetes based on model 1. Model 3: Further adjusted for HDL-C, SCr and BUN based on model 2. The proportional hazards assumption was tested using Schoenfeld residuals and confirmed via the global Schoenfeld test ($P > 0.05$ for all models). E-values were computed to estimate the influence of unmeasured confounding on the associations. Variance inflation factors (VIFs) were calculated to assess multicollinearity among covariates, and variables with VIF > 5 were excluded from the analysis. Cumulative

incidence of diabetes over time was estimated using Kaplan-Meier survival curves, stratified by quartiles of ALT, AST, and the ALT/AST ratio. Log-rank tests were performed to compare survival distributions across quartiles. To assess the predictive performance of hepatic biomarkers for incident diabetes, time-dependent receiver operating characteristic (ROC) analyses were conducted at 3, 4, and 5 years of follow-up, and area under the curve (AUC) values were calculated. In addition, subgroup analysis (including age, gender, BMI, and family history of diabetes) was performed to explore other factors that affect the association of hepatic biomarkers with diabetes incidence.

Finally, optimal cutoff values for hepatic biomarkers, and TyG index were determined based on the maximum selected rank statistic. The synergistic effects of hepatic biomarkers and TyG index on diabetes risk were evaluated using joint association. Mediation analysis was carried out to investigate the role of the TyG index in the association between hepatic biomarkers and diabetes risk. The proportion of mediation was estimated using R software with bootstrapping (1000 iterations) to derive 95% CIs. Additionally, to assess robustness to unmeasured confounding, the present study calculated E-values for the total, direct, and indirect effects, indicating the required strength of an unmeasured confounder's association with both exposure and outcome to fully explain away the observed effect. All tests used two-tailed $P < 0.05$.

Results

Study populations

The study cohort comprised a total of 50463 participants, with a mean age of 44.41 ± 13.24 years (**Table 1**). The gender distribution showed a majority of males, accounting for 55.73% (N = 28122) of the cohort, while females constituted the remaining 44.27% (N = 22341). Throughout the observational phase (mean 3.08 years), 1309 participants (2.59%) developed diabetes, while the majority (N = 49154, 97.41%) remained No-diabetes. Participants who developed diabetes were older than those in the No-diabetes group (57.05 ± 12.28 years vs. 44.07 ± 13.10 years, *P* <0.001). There was a higher proportion of males in the diabetes group (67.15% vs. 55.42%, *P* <0.001). Liver enzyme profiles were markedly elevated in the diabetes group. ALT levels were higher (median: 25.00 U/L vs. 18.00 U/L, *P* <0.001), as were AST levels (median, 25.00 U/L vs. 22.00 U/L, *P* <0.001). The ALT/AST ratio was also significantly increased in the diabetes group (median: 1.00 vs. 0.84, *P* <0.001). Insulin resistance, as measured by the TyG index, was substantially higher in the diabetes group (mean ± SD: 9.00 ± 0.60 vs. 8.41 ± 0.61, *P* <0.001). Other noteworthy differences included higher FPG, BMI, DBP, SBP, TG, LDL-C, SCr, TC, BUN, and lower HDL-C (all *P* <0.001). Additionally, Family history of diabetes, current drinking and smoking appeared more prevalent in the diabetes group (all *P* <0.001).

Association of hepatic biomarkers with diabetes

The Kaplan-Meier graphs depict the cumulative diabetes incidence over time, categorized by quartiles (Q1-Q4) of ALT, AST, and the ALT/AST ratio. Participants

31 were classified into quartiles according to baseline biomarker levels, with Q1 denoting the lowest quartile and Q4 indicating the highest quartile. The log-rank tests for all three biomarkers were statistically significant (Log-rank $P < 0.0001$), confirming that high ALT, AST, and the ALT/AST ratio were associated with a higher incidence of diabetes (**Figure 2**).

In **Supplementary Table 2,** the present study showed the results of univariate COX regression analysis. Since the association between ever smoking (compared with current smoking) and diabetes did not show statistical significance in univariate COX regression, smoking status was not included as an adjustment variable in subsequent adjustment models. In addition, the present study performed collinearity analysis on various risk factor variables for diabetes and found that the VIF of TC was 5.4, showing high collinearity, and the VIF of LDL was 4.9, close to 5. Therefore, TC and LDL were also excluded from the multivariate analysis (**Supplementary Figure 1**).

Table 2 displays the results of multivariate analyses, demonstrating robust and independent associations between ALT, AST, and the ALT/AST ratio and the risk of diabetes, with the ALT/AST ratio exhibiting the most notable effect. For every unit increase in ALT and AST, the risk of diabetes rose by 1% in all adjusted models (ALT: HR = 1.01; 95% CI: 1.00-1.01, $P < 0.001$; AST: HR = 1.01; 95% CI: 1.00-1.01, $P < 0.001$). When examined by quartiles, ALT demonstrated a clear dose-response relationship (P for trend < 0.001), the highest quartile (Q4) associated with a more than

twofold increase in diabetes risk compared to the lowest quartile (Q1) (HR = 2.28; 95% CI: 1.87-2.77, $P < 0.001$). In contrast, the association between AST quartiles and diabetes risk did not reach statistical significance. Compared with ALT and AST, the ALT/AST ratio merged as an even stronger predictor of diabetes risk. A unit increase in the ALT/AST ratio raised diabetes risk by 4% across all models (HR = 1.04; 95% CI: 1.02-1.05, $P < 0.001$). When analyzed by quartiles, the highest quartile (Q4) of the ALT/AST ratio demonstrated a nearly threefold increase in diabetes risk compared to the lowest quartile (Q1) (HR = 2.94; 95% CI: 2.42-3.57, $P < 0.001$), with a clear and strong dose-response trend (P for trend < 0.001). This suggests that the ALT/AST ratio, which integrates both ALT and AST levels, may provide superior risk stratification compared to either enzyme alone.

Finally, the proportional hazard assumption of the fully adjusted model (model 3) was tested and confirmed through the global Schoenfeld test (ALT: $P = 0.487$; AST: $P = 0.416$; the ALT/AST ratio: $P = 0.145$), which confirmed that the assumption was not violated (**Supplementary Figure 2-4**). Additionally, to evaluate the potential influence of unmeasured confounders on the associations between ALT, AST, and the ALT/AST ratio with diabetes risk, E values were calculated. The computed E values for ALT, AST, and the ALT/AST ratio were 1.11, 1.11, and 1.24, respectively, suggesting minimal impact of unmeasured confounders on these associations.

Subgroup analysis showed that the relationship of ALT, AST, and the ALT/AST ratio

with diabetes risk remained consistent across various specific groups, although there was an interaction between sex and ALT/AST levels regarding diabetes risk (**Supplementary Table 3**).

Predictive performance of hepatic biomarkers for diabetes

15 In the original study, individuals who developed diabetes during the 2-year follow-up period were excluded from the analysis. Additionally, in the present study, approximately 98% of the study population completed follow-up within 5 years. Therefore, the present study performed time-dependent ROC analyses at three time points, 3, 4, and 5 years, to evaluate the predictive performance of ALT, AST, and the ALT/AST ratio for incident diabetes. As shown in **Figure 3**, the AUC values of ALT, AST, and the ALT/AST ratio for identifying diabetes fluctuated between 0.59 and 0.64, with Youden index fluctuating between 0.17 and 0.22 (**Supplementary Table 4**), suggesting that ALT, AST, and the ALT/AST ratio have a certain predictive value in assessing future diabetes risk (**Supplementary Figure 5**).

Joint association of hepatic biomarkers, and TyG index with diabetes

As shown in **Supplementary Table 5**, there were robust and independent associations between ALT, AST, and the ALT/AST ratio and the TyG index. This suggests that elevated liver enzyme levels increase insulin resistance. TyG index had good predictive value for future diabetes occurrence (**Supplementary Table 4 and Supplementary Figure 5**).

Additionally, the present study attempted to conduct a joint analysis of liver enzyme profile, TyG index, and diabetes. Based on the maximum selected rank statistic, the optimal cutoff values for the most significant association of ALT, AST, the ALT/AST ratio, and TyG index with diabetes were determined to be 30.3 U/L, 27.9 U/L, 1.02, and 8.78, respectively (**Supplementary Figure 5-8**). The results of the joint analysis showed that there was a synergistic effect between elevated liver enzyme levels and a higher TyG index, which significantly increased the risk of diabetes. Specifically, individuals with ALT levels >30.3 U/L and a TyG index >8.78 had a 4.79-fold increased risk of diabetes compared to those with lower ALT and TyG index (Model 3: HR = 4.79; 95% CI: 4.06-5.65, $P < 0.001$). Similarly, individuals with AST levels >27.9 U/L and a TyG index >8.78 had a 3.69-fold increased risk of diabetes compared to the reference group (Model 3: HR = 3.69; 95% CI: 3.13-4.35, $P < 0.001$). Those with an ALT/AST ratio > 1.02 and a TyG index > 8.78 also displayed a higher risk (Model 3: HR = 5.23; 95% CI: 4.42-6.18, $P < 0.001$) (**Table 3**).

Mediation analysis of TyG index effects hepatic enzyme association with diabetes

The mediation analysis yielded compelling evidence of the TyG index's pivotal role in mediating the hepatic enzyme (ALT, AST, and the ALT/AST ratio)-diabetes association (**Table 4**). TyG index mediates 40.25% (95% CI: 31.13%-43.52%) of the total effect of ALT on diabetes risk, establishing its substantial contribution to this pathway. Similarly, the TyG index accounted for 36.45% (95% CI: 28.74%-52.14%) of the total effect of

AST on diabetes risk. Notably, the ALT/AST ratio demonstrated the most pronounced mediated effect, with the TyG index explaining 76.97% (95% CI: 19.84%-78.61%) of its total effect on diabetes risk. Additionally, the present study calculated the E values for the total effect (exposure → outcome), direct effect (exposure → outcome after adjusting for mediating variables), and indirect effect (mediated by the TyG index) (**Supplementary Table 5)**. The E value for ALT (total effect) was 1.10, and the E value for indirect effect mediated by TyG was 1.06. The E value for AST (total effect) was 1.10, and the E value for indirect effect mediated by TyG was 1.06. The E value for the ALT/AST ratio (total effect) was 1.49, and the E value for the indirect effect mediated by TyG was 1.41 (**Supplementary Table 7**). These results suggest that unmeasured confounding factors had a negligible impact on the mediation analysis. These robust findings elucidated the intricate relationship between hepatic biomarkers and diabetes pathogenesis through the lens of TyG index mediation.

Discussion

This investigation establishes hepatic biomarkers-ALT, AST, and the ALT/AST ratio-as reliable predictors of incident diabetes within a Chinese adult cohort and underscores the TyG index's pivotal mediating influence on the association between elevated liver enzymes and diabetes risk. These findings offer new insights into the mechanistic pathways linking hepatic dysfunction to diabetes pathogenesis.

Hepatic Biomarkers and Diabetes Risk

In addition to confirming previously established associations, this analysis highlights the ALT/AST ratio as a superior predictor of diabetes risk, expanding upon existing evidence. For instance, in a cohort study of 7963 multiethnic Chinese individuals, Wang et al. examined the relationship between hepatic biomarkers-γ-glutamyl transpeptidase (GGT), ALT, alkaline phosphatase (ALP), and AST-and diabetes risk. Their analysis revealed that only ALT (OR = 1.18, 95% CI: 1.09, 1.28) and GGT (OR = 1.18, 95% CI: 1.12, 1.25) showed a significant correlation with an elevated diabetes risk [18]. Liu et al. demonstrated that individuals with elevated ALT levels had a diabetes prevalence 2.99 times higher than those with normal ALT levels [19]. However, the combined ALT/AST ratio has been underexplored as a predictor of incident diabetes. Consistent with the present study's findings, Wang et al. showed that an elevated ALT/AST ratio was independently linked to insulin resistance among U.S. adults, highlighting its potential as a metabolic marker [20]. Similarly, Yan et al. observed an increased risk of cardiometabolic outcomes associated with higher ALT/AST ratios, though this study did not focus on incident diabetes [21]. The findings of the present study extend these observations by demonstrating the predictive value of the ALT/AST ratio for diabetes risk in a large East Asian cohort.

The biological mechanisms underlying the ALT/AST ratio’s superiority as a predictor of diabetes risk are likely multifactorial. First, the ALT/AST ratio’s association with hepatic fat accumulation aligns with recent findings in metabolic liver disease. Plasma aldosterone concentration, a mediator of hepatic fibrosis, independently correlated with

MAFLD prevalence in hypertensive patients, further supporting the role of hepatic stress in metabolic dysregulation [22]. Similarly, Shen et al. identified systemic inflammation as a shared pathway linking elevated liver enzymes to MAFLD [23]. Therefore, hepatic fat accumulation and inflammation reflected by the ALT/AST ratio might be key contributors to systemic insulin resistance [24, 25]. Additionally, studies have shown that hepatic steatosis, characterized by elevated ALT relative to AST, is strongly associated with impaired glucose metabolism and β-cell dysfunction[26, 27]. In contrast, isolated elevations in AST may reflect extrahepatic processes such as muscle damage or hemolysis, which are less directly linked to metabolic dysregulation [28]. Therefore, the ALT/AST ratio, integrating both ALT and AST levels, may serve as a more comprehensive marker of hepatic insulin sensitivity than either enzyme alone, consistent with prior studies [11, 29]. Additionally, Emerging research on the liver-pancreas axis provides further mechanistic insights. The liver communicates with pancreatic β-cells via hepatokines (e.g., fetuin-A, selenoprotein P) and inflammatory cytokines (e.g., IL-6, TNF-α) that are elevated in hepatic steatosis [30-32]. For instance, fetuin-A, secreted by fatty liver, impairs insulin secretion by downregulating pancreatic β-cell IRS2 expression, while hepatic IL-6 exacerbates systemic inflammation and β-cell apoptosis. These pathways are amplified when the ALT/AST ratio is elevated, reflecting hepatic metabolic stress. Notably, the TyG index-mediating ~77% of the ALT/AST ratio’s effect-may serve as a surrogate for this crosstalk, as it correlates with both hepatic insulin resistance and impaired β-cell compensation.

Notably, the observed risk reduction in AST Q2-Q3 (HR≈0.83) might reflect the dual role of AST in metabolic and non-hepatic pathways in the present study. Unlike ALT (primarily hepatic), AST is also expressed in muscle and erythrocytes. Moderate AST elevations (Q2-Q3) may indicate enhanced mitochondrial activity in peripheral tissues (e.g., skeletal muscle), which improves glucose oxidation and insulin sensitivity [28, 33]. Experimental studies suggest AST-containing tissues may buffer systemic oxidative stress during early metabolic dysfunction, potentially delaying diabetes onset [34]. Additionally, the associations of ALT (*P*-interaction=0.002) and AST (*P*-interaction=0.002) with diabetes risk were stronger in females than males, despite males having higher baseline enzyme levels. This aligns with studies suggesting that hepatic steatosis more severely impacts glucose metabolism in women due to estrogen's modulation of hepatic insulin sensitivity [35]. The sex interaction might also reflect hormonal regulation of hepatic lipid metabolism. For example, estrogen deficiency in postmenopausal women exacerbates hepatic insulin resistance, amplifying the ALT-diabetes link [36]. Additionally, while the AUC values for ALT (0.63–0.64), AST (0.59–0.64), and the ALT/AST ratio (0.59–0.63) indicate modest discriminative ability for incident diabetes. However, in primary care, these low-cost indicators can be used to prioritize high-risk individuals. For example, those with persistently elevated ALT levels might need to be re-examined for diabetes within 2 years. Additionally, the synergistic effect with the TyG index supported its use as a component of composite risk assessment.

Association of the TyG Index and Hepatic Biomarkers with diabetes Risk

The joint analysis revealed significant synergistic effects between elevated hepatic biomarkers and the TyG index. Notably, the combination of a high ALT/AST ratio and elevated TyG index conferred the greatest risk of diabetes, with a hazard ratio of 5.23. This synergistic effect suggests that the interplay between hepatic dysfunction and insulin resistance may amplify the risk of diabetes, a finding consistent with recent studies emphasizing the importance of integrating hepatic and metabolic profiles in diabetes risk assessment [11, 12].

The synergistic effect observed between elevated hepatic biomarkers and the TyG index underscores the interplay between hepatic dysfunction and systemic insulin resistance in driving diabetes risk. The TyG index integrates fasting glucose and TG levels and has been increasingly recognized as a predictor of cardiometabolic outcomes [37]. The joint analysis of the present study revealed that individuals with elevated liver enzymes and a high TyG index represent a particularly high-risk subgroup, with the combination of a high ALT/AST ratio and elevated TyG index conferring the greatest hazard ratio of 5.23. This suggests that integrating hepatic and metabolic profiles enhances diabetes risk prediction, supporting the growing emphasis on multifactorial risk assessment in clinical practice.

The present study also offers new evidence for the TyG index’s mediating effect on the hepatic biomarker-diabetes pathway. This analysis revealed that the TyG index

accounted for 40.25%, 36.45%, and 76.97% of the total effects of ALT, AST, and the ALT/AST ratio, respectively, on diabetes risk. As a widely used insulin resistance surrogate, the TyG index has been shown to predict diabetes risk in diverse populations, including Asian cohorts [38-40]. However, its role as a mediator between hepatic biomarkers and diabetes risk has not been thoroughly explored. The findings of the present study align with mechanistic studies indicating that hepatic inflammation and insulin resistance are closely intertwined [41]. Elevated hepatic biomarkers, particularly ALT, are associated with systemic inflammation and oxidative stress, which impair insulin signaling and elevate the TyG index [34, 42]. This suggests that the TyG index may serve as a bridge between hepatic dysfunction and impaired glucose metabolism. However, unlike prior studies, this analysis provides quantitative evidence of the TyG index’s mediating effect, highlighting its contribution in the pathogenesis of diabetes associated with liver dysfunction. However, although the mediation analysis accounted for established confounders, the possibility of residual confounding due to unmeasured variables (e.g., dietary factors, genetic influences) remains.

Clinical Implications

The findings of this study have significant clinical implications. The incorporation of hepatic biomarkers, especially the ALT/AST ratio, along with the TyG index, into standard screening procedures could improve the early detection of individuals predisposed to diabetes. This approach would enable the timely initiation of personalized interventions, including lifestyle modifications, weight management, and pharmacological treatments aimed at improving insulin sensitivity. For individuals with

elevated hepatic biomarkers and a high TyG index, weight loss and dietary changes might be recommended to reduce hepatic fat and improve insulin sensitivity. Given the TyG index's strong mediation effect, targeting triglyceride and glucose metabolism (e.g., increasing omega-3 fatty acids, reducing refined carbohydrates) might be especially beneficial. Metformin, which ameliorates hepatic insulin resistance and lowers liver enzymes, could be considered for high-risk patients with both elevated hepatic biomarkers and TyG index, even before diabetes onset.

Strengths and Limitations

This study is strengthened by its large, well-characterized cohort, rigorous statistical adjustments, and in-depth mediation analysis. However, several limitations should be noted. First, while the mediation analysis suggests a potential causal pathway from hepatic biomarkers to diabetes via the TyG index, residual confounding—such as the effect of skeletal muscle mass on AST levels—could still exist. Future Mendelian randomization studies are needed to confirm causality. Second, the reliance on baseline measurements of hepatic biomarkers and the TyG index might not fully capture their dynamic changes over time, which could influence the observed associations. Third, the dataset lacked information on key comorbidities (e.g., non-alcoholic fatty liver disease, cardiovascular diseases) that may concurrently alter hepatic enzyme levels and insulin resistance. Recent studies highlight MAFLD as a critical confounder in liver enzyme-metabolic disease relationships [23]. Although the present study adjusted for available metabolic confounders (BMI, blood pressure, lipids), this unmeasured

variability could partially bias the results. Fourth, this analysis lacked adjustment for glucose-lowering medications (e.g., metformin, insulin), lipid-lowering drugs (e.g., statins), and antihypertensive medications (e.g., ACE inhibitors, beta-blockers), which may influence hepatic enzyme levels and metabolic outcomes. However, excluding participants with baseline diabetes minimized confounding from glucose-lowering medications, and Model 3 adjusted for SBP/DBP and lipid parameters (HDL-C and TG), which are linked to medication use and partially mitigate their effects. Lastly, as the study cohort consisted solely of Chinese adults, the findings may have limited applicability to populations with different metabolic characteristics.

Conclusion

In conclusion, hepatic biomarkers, especially the ALT/AST ratio, have a strong association with a high incidence of diabetes, and the TyG index was a key mediator in this relationship. These findings underscore the value of incorporating hepatic and metabolic profiles into diabetes risk assessment and highlight the potential for targeting insulin resistance to reduce diabetes risk in individuals with elevated liver enzymes. Future research could explore the utility of these markers in diverse populations and assess whether interventions targeting hepatic dysfunction and insulin resistance can reduce the incidence of diabetes.
