# Supplementary material for: Association of hepatic biomarkers with incident diabetes: a mediation analysis of the triglyceride-glucose index in a large Chinese cohort
Source: Lipids Health Dis. 2025 Jul 21;24:246. doi: 10.1186/s12944-025-02661-z (PMC12278604; doi:10.1186/s12944-025-02661-z)
Supplement: Supplementary file 3 — Supplementary Material 3 [file 12944_2025_2661_MOESM3_ESM.pdf]

Bullet Edits

EDITORIAL CERTIFICATE

This document certifies that the paper listed below was edited and proofread for proper English language, grammar, punctuation, spelling, and overall style by one or more than one highly qualified native speakers at Bullet Edits. All of the suggested amendments were tracked with the Microsoft Word "Track Changes" feature. Therefore, the author had the option to reject or accept each change individually.

Manuscript Title:

Association of Hepatic Biomarkers with Incident Diabetes: A Mediation Analysis of the Triglyceride-Glucose Index in a Large Chinese Cohort

Anti-counterfeiting Code:

bf7970d482a49c5a0391c6907eab0c97

Date Issued:

2025-06-26 18:12:19

Bullet Edits is a registered company headquartered in the UK with a global presence. We offer a range of editing, proofreading services to authors. Our Ph.D. editors are all native English speakers from the USA and UK. Authors who work with Bullet Edits are guaranteed excellent language quality and timely delivery.

Address: BULLET EDITS LIMITED, 85 Great Portland Street, London,UK
Tel: UK. (+44)20457 70286 / US.(+1)312-313-9179
Web: www.bulletedits.cn/Email: info@bulletedits.cn
VAT: GB 378 9316 43

BULLET EDITS LIMITED ★ COMPANY NO. 13345261 SEAL ★ ENGLAND AND WALES
